# GLUT-1 as a predictor of worse prognosis in pancreatic adenocarcinoma: immunohistochemistry study showing the correlation between expression and survival

**DOI:** 10.1186/s12885-020-07409-9

**Published:** 2020-09-23

**Authors:** Mar Achalandabaso Boira, Marcello Di Martino, Carlos Gordillo, Magdalena Adrados, Elena Martín-Pérez

**Affiliations:** 1 Division of Hepatobiliary Pancreatic Surgery, Hospital Universitario de La Princesa, 28006 Madrid, Spain; 2 Pathology Department, Hospital Universitario de La Princesa, 28006 Madrid, Spain

**Keywords:** GLUT-1, Antibody, Pancreatic cancer, Prognostic factor

## Abstract

**Background:**

Various parameters have been considered for predicting survival in pancreatic ductal adenocarcinoma. Information about western population is missing. The aim of this study is to assess the association between Glucose transporter type 1 (GLUT-1) expression and prognosis for patients with PDAC submitted for surgical resection in a European cohort.

**Methods:**

Retrospective analysis of PDAC specimens after pancreatoduodenectomy assessing GLUT-1 expression according to intensity (weak vs strong) and extension (low if < 80% cells were stained, high if > 80%) was performed. Statistical analysis was performed using the exact Fisher test, Student t test or the Mann-Whitney U test. Survival was analysed using the Kaplan-Meier method and compared with the Log-rank test. The differences were considered significant at a two-sided *p* value of < 0.05. All statistical analyses were performed using SPSS® 23.0 for Windows (SPSS Inc., Chicago, IL, USA).

**Results:**

Our study consisted of 39 patients of which 58.9% presented with weak and 41.1% with strong intensity. The median extension was 90%: 28.2% cases presented with a low extension and 71.8% with a high extension. No significant differences related to intensity were found. The high-extension group showed a higher percentage of T3 PDAC (92.9% vs 63.6%, *p* = 0.042) and LNR20 (35.7% vs 0%, *p* = 0.037) as well as shorter disease-free survival (17.58 vs 54.46 months; *p* = 0.048).

**Conclusions:**

Our findings suggest that GLUT-1 could be related to higher aggressivity in PDAC and could be used as a prognostic marker, identifying patients with a worse response to current therapies who could benefit from more aggressive treatments.

## Background

Pancreatic carcinoma is one of the most aggressive tumours in humans and is the fourth greatest cause of cancer mortality in Europe [1]. Surgical resection is the only chance of cure and long-term survival in resectable tumours [2]. However, only 15–20% of patients with carcinoma of the pancreas are subsidiaries of curative resection and five-year survival remains low, compared with other digestive tumours [3]. In patients undergoing resection, various clinical and pathological prognostic parameters such as tumour type and size, lymph node involvement, the radicality of resection, degree of differentiation, lymphatic, vascular or perineural infiltration, and specific oncogene mutations have been considered as prognostic factors [4–6]. In recent years, new molecular prognostic markers have been investigated to optimise the reliability of prognostic information and select subgroups of patients who could benefit from specific treatment algorithms. An example of these are mutations in breast cancer genes and greater sensitivity to combinations with chemotherapy [7, 8]. The Glucose Transporter type 1 (GLUT-1) is one of 14 in a family of facilitators of glucose transport in mammals; it is one of the most ubiquitously distributed subtypes. They are passive transporters that use an independent energy system to transport glucose through concentration gradients. Their expression and activity are regulated by growth factors and oncogenes.

Numerous studies have shown that GLUT-1 overexpression in tumours from different sites can suggest a worse prognosis, such as in colorectal cancer [9], lung cancer [10], mesothelioma [11], head and neck carcinoma [12], ovarian cancer [13], urothelial carcinoma [14], prostate cancer [15], esophagogastric cancer [16, 17] and sarcomas [18]. The existing data on the prognostic significance of the overexpression of GLUT-1 in pancreatic ductal adenocarcinoma (PDAC) has been limited and not consistent, having increased exponentially in recent years with positive results. The current study aims to explore the association between GLUT-1 expression and prognosis in a European cohort of patients undergoing pancreaticoduodenectomy (PD) for PDAC after a long-term follow-up.

## Methods

We conducted a retrospective cohort study of patients diagnosed with PDAC undergoing PD in our centre during the period 2006 to 2013 with subsequent follow-ups until November 2018.

### Inclusion criteria

Consecutive patients undergoing PD with a diagnosis of adenocarcinoma of the head of the pancreas, who had sufficient histological material to make the stains, were included. Patients older than 18 years old, in whom a multislice computed tomography (CT) was diagnostic of resectable PDAC, who had not received preoperative chemotherapy or radiotherapy, and who had an American Society of Anaesthesiologist classification (ASA) of I, II, or III, were included. Patients with unresectable intraoperative pancreatic tumours, in whom surgical resection was not performed or whose final pathology was not adenocarcinoma were excluded.

### Demographic and staging parameters

For each patient, the following data were extracted from the institutional pancreas database: demographic parameters (age, gender, body mass index [BMI], ASA, comorbidities and symptoms at diagnosis), postoperative complications (reported as per the Clavien-Dindo classification [19]), staging parameters (tumour size [T], lymph node status [N], lymph node ratio 20 [LNR20], defined as the ratio between affected and total retrieved lymph nodes being higher than 20%, tumour differentiation, stage, vascular, perineural and lymphatic invasion, positive resection margin rate [R] and recurrence), and survival parameters (disease-free survival [DFS] and overall survival [OS], measured from surgical intervention to recurrence or death respectively).

### Resectability criteria

For all patients included in the study, prior to surgical intervention, the resectability of the tumour was assessed during the Multidisciplinary Team Meeting of Digestive Surgery at our centre. All the patients included in the study underwent a 64-slice CT (Siemens Somaton Sensation 64® - Siemens Healthcare© - Erlangen, Germany). The lesions were considered resectable in the absence of extended disease or vascular infiltration (presence of fatty planes around the vessels) or contact with the superior mesenteric vein or portal vein ≤180° in the absence of vein contour irregularity [20].

### Surgical technique

All patients underwent a classic Whipple procedure performed by experienced pancreatic surgeons. End-to-side duct-to-mucosa pancreaticojejunostomy or dunking pancreaticojejunostomy was performed at the surgeons’ discretion, depending on the pancreas consistency. End-to-side hepaticojejunostomy and transmesocolic end-to-side, double layer gastrojejunostomy was the standard technique used in our unit.

### Staining protocol

Each surgical piece was assessed with a ultraView Universal DAB v1.02.0018 detection kit (Ventana Medical Systems, Tucson, Arizona). Olympus BX43F microscope with 40, 100, 200 and 400 type of objective lenses was used. The images were obtained with Olympus UC90 microscope camera, with acquisition software Olympus cellSens Entry 1.18*.* For diagnostic purposes, 5 μm-thick paraffin sections were stained with hematoxylin and eosin. All the histological preparations were reviewed by two pathologists without previous knowledge of the prognostic factors and/or clinical evolution. Our GLUT-1 protocol consisted of heating the slides to 65 °C and incubating them for 12 min in a stove. Afterwards, dewaxing was carried out at 72 °C for 8 min with xylene, 99° ethanol and 96 °C ethyl. Then cellular conditioning was performed at 95 °C for 8 min. The buffer was subsequently washed at 36° for 4 min. After washing, the sections were incubated with a drop of UV INHIBITOR (cell inhibitor) for 4 min at 36 °C, then with a drop of GLUT-1 (rabbit polyclonal antibody, Roche) for 32 min and afterwards with a drop of universal multimeric antibody for 8 min. Next, a drop of chromogen diaminobenzidine was used as the substrate in the colour development reaction and incubated for 8 min. The sections were then counterstained with hematoxylin and incubated for 12 min. Finally, a drop of Bluing reagent was applied to the counterstained tile and incubated for 4 min.

### Pathological analysis

A sample corresponding to the surgical specimen after PD of each patient was analysed by two independent investigators who were unaware of the outcome of the patients. The expression of GLUT-1 was considered positive when it was present in the membrane, the cytoplasm, or both. The erythrocytes and perineurium were considered as internal positive controls for GLUT-1 staining. Each preparation was evaluated at 40, 100, and 400 magnification. The staining was evaluated by two parameters: intensity and extension. The intensity was classified as weak or strong, defining strong as the same intensity as that of the positive controls; the extension was defined by the percentage of cells that presented the strongest staining (cytoplasmic, membranous, or both), which were subsequently grouped in low extension (< 80% of cells stained) versus high extension (≥80% of cells stained), thus identifying two groups for later comparison as reported previously in the literature.

### Statistical analysis

Descriptive data were expressed as counts and proportions for categorical variables; continuous variables were presented as a median within an interquartile range. Statistical analysis was performed using the exact Fisher test for the comparison of categorical variables and the Student t test or the Mann-Whitney U test for continuous variables. Survival was analysed using the Kaplan-Meier method and compared with the Log-rank test. The differences were considered significant at a two-sided *p* value of < 0.05. All statistical analyses were performed using SPSS® 23.0 for Windows (SPSS Inc., Chicago, IL, USA).

## Results

### Demographic and surgical parameters

Our study sample consisted of 39 patients with an average age of 68.4 years. The distribution by sex corresponded to 23 men and 16 women. The rest of the data are included in Table 1. End-to-side duct-to-mucosa pancreaticojejunostomy anastomosis was performed on 35 patients and dunking on four. The median surgical time was 420 min (360–535 min) and the median blood loss was 350 ml (200–612 ml). Regarding complications data, 17 patients had an uneventful postoperative course, 11 patients had Clavien-Dindo complications graded < 3, and 11 patients graded ≥3. There was a case of reoperation with subsequent death. Thirty patients presented recurrence, with the most frequent location being the liver with 13 patients (33.3%). The median follow-up was 16 months (9.7–39.2 months) and 25% of the patients had a follow-up longer than 40 months. The median DFS was 8 months (5–20.5 months); 32 patients died during the follow-up. The median OS was 16 months (9.7–39.2 months).

**Table 1:** Clinicopathological parameters of patients with pancreatic adenocarcinoma

Characteristics	Patients
Age, years	
Mean	68.49 years ±11.59
Range	40–93
Gender	
Male	23 (58.9%)
Female	16 (41.1%)
BMI	
Mean	26.54 ± 5.155
Range	17.30–36.03
ASA	
I	1 (2.6%)
II	24 (61.5%)
III	12 (30.8%)
IV	1 (2.6%)
Comorbidities	
Hypertension	15 (38.5%)
Diabetes Mellitus	8 (20.5%)
Dislipemia	6 (15.4%)
Smoker	16 (41%)
Alcohol excess	8 (20.5%)
Chronic pancreatitis	1 (2.6%)
Other tumors in the past	2 (5.1%)
Symptoms	
Jaundice	33 (84.6%)
Abdominal pain	7 (17.9%)
Non induced weight loss	16 (41%)
Nausea and vomiting	3 (7%)
Pruritus	6 (15.4%)
Fever of tumoral origin	1 (2.6%)
Asthenia	1 (2.6%)
Size	
T1	1 (2.6%)
T2	5 (12.8%)
T3	33 (84.6%)
Lymph node	
N0	18 (46.2%)
N1	21 (53.8%)
Differentiation	
Good	8 (20.5%)
Moderate	20 (51.3%)
Poor	10 (25.6%)
Stage	
IA	1 (2.6%)
IB	3 (7.7%)
IIA	15 (38.5%)
IIB	20 (51.3%)

### Staining parameters

**Fig. 1:**
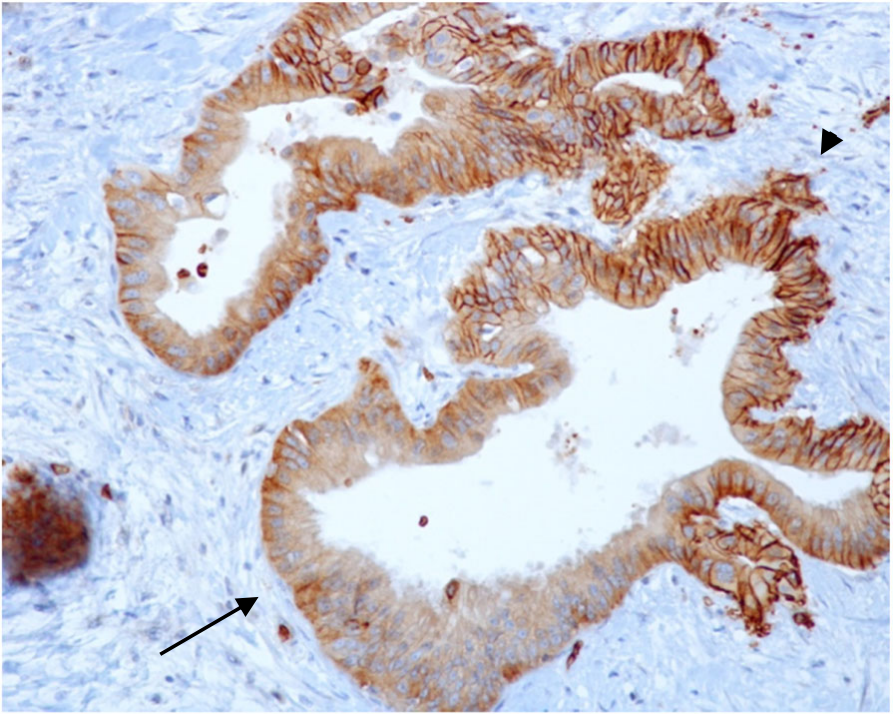
GLUT-1 stained head of the pancreas adenocarcinoma sample. Visualized at 200 magnification, showing weak staining (arrow) and strong staining (arrowhead)

GLUT-1 was overexpressed in all pancreatic cancer samples while there was no expression in adjacent normal pancreatic tissue. According to the intensity of the samples, 23 patients presented weak staining (58.9%) and 16 presented strong staining (41.1%). With regards to extension, the median was 90%, 11 patients (28.2%) had low-extension (< 80%) and 28 patients (71.8%) had high-extension (Fig. 1).

### Comparison of GLUT-1 intensity with prognostic variables and survival analysis

**Fig. 2:**
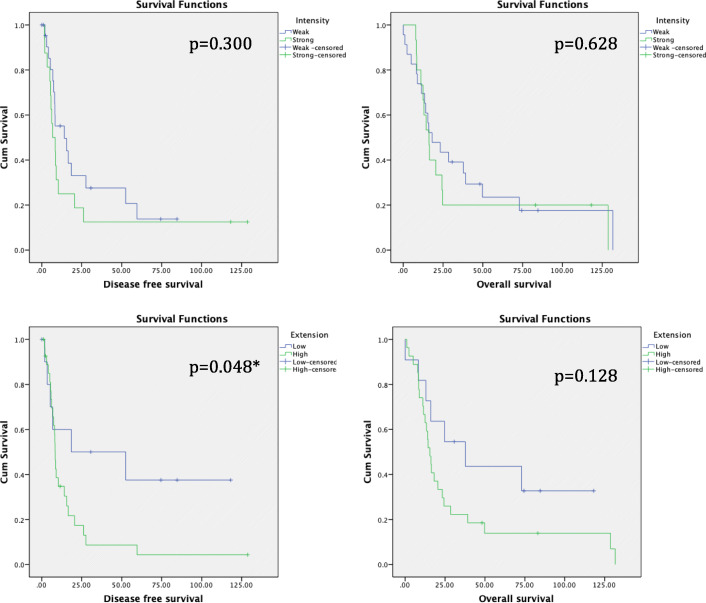
Comparison of GLUT-1 intensity and extension and survival analysis

Patients were divided in weak versus strong intensity (same as the positive controls) for comparison purposes. No significant differences between the groups were found; nevertheless, strong staining seemed to present a higher rate of N+ (62.5% vs 47.8%, *p* = 0.516), worse tumour differentiation (43.8% vs 13.6%, *p* = 0.062), higher rate of R1 (62.5% vs 47.8%, p = 0.516), and higher recurrence rate (87.5% vs 69.6%, *p* = 0.262) as shown in Table 2. Regarding survival analysis, the results did not reach statistical significance, although both DFS and OS were shorter in the strong intensity group (23.54 vs 27.14 months, *p* = 0.300 and 37.57 vs 41.36 months, *p* = 0.628) (Fig. 2).

**Table 2:** Association between GLUT-1 intensity and extension and clinicopathological variables in pancreatic cancer patients

**Patient characteristics**		**Weak intensity** ***n*** **= 23**	**Strong intensity** ***n*** **= 16**	***P*** **value**
Age	< 60	5 (21.7%)	2 (12.5%)	.678
	> 60	18 (78.3%)	14 (87.5%)	
Gender	Male	14 (60.9%)	9 (56.3%)	1
	Female	9 (39.1%)	7 (43.8%)	
T	T1-T2	4 (17.4%)	2 (12.5%)	1
	T3	19 (82.6%)	14 (87.5%)	
N	N0	12 (52.2%)	6 (37.5%)	.516
	N1	11 (47.8%)	10 (62.5%)	
Differentiation	Good	19 (86.4%)	9 (56.3%)	**.**062
	Poor	3 (13.6%)	7 (43.8%)	
Microvascular invasion	No	0	0	1
	Yes	9 (100%)	11 (100%)	
Lymphatic invasion	No	9 (45%)	5 (31.3%)	.501
	Yes	11 (55%)	11 (68.8%)	
Perineural invasion	No	3 (15%)	2 (12.5%)	1
	Yes	17 (85%)	14 (87.5%)	
LNR 20	No	4 (17.4%)	6 (37.5%)	.264
	Yes	19 (82.6%)	10 (62.5%)	
Resection margin	R0	12 (52.2%)	6 (37.5%)	.516
	R1	11 (47.8%)	10 (62.5%)	
Complications Clavien-D	< 3	14 (66.7%)	12 (75%)	.723
	> 3	7 (33.3%)	4 (25%)	
Recurrence	No	7 (30.4%)	2 (12.5%)	.262
	Yes	16 (69.6%)	14 (87.5%)	
DFS (m)		27.14	23.54	.300
OS(m)		41.36	37.57	.628
**Patient characteristics**		**Low extension** ***n*** **= 11**	**High extension** ***n*** **= 28**	***P*** **value**
Age	< 60	2 (18.2%)	5 (17.9%)	1
	> 60	9 (81.8%)	23 (82.1%)	
Gender	Male	8 (72.7%)	15 (53.6%)	.471
	Female	3 (27.3%)	13 (46.4%)	
T	T1-T2	4 (36.4%)	2 (7.1%)	.042*
	T3	7 (63.6%)	26 (92.9%)	
N	N0	8 (72.7%)	10 (35.7%)	.072
	N1	3 (27.3%)	18 (64.3%)	
Differentiation	Good	20 (83.3%)	8 (57.1%)	1
	Poor	4 (16.7%)	6 (42.9%)	
Microvascular invasion	No	0	0	1
	Yes	8 (100%)	12 (100%)	
Lymphatic invasion	No	4 (40%)	10 (38.5%)	1
	Yes	6 (60%)	16 (61.5%)	
Perineural invasion	No	2 (20%)	3 (11.5%)	.603
	Yes	8 (80%)	23 (88.5%)	
LNR 20	No	11 (100%)	18 (64.3%)	.037*
	Yes	0	10 (35.7%)	
Resection margin	R0	5 (45.5%)	13 (46.4%)	1
	R1	6 (54.5%)	15 (53.6%)	
Complications Clavien-D	< 3	8 (72.7%)	18 (69.2%)	1
	> 3	3 (27.3%)	8 (30.8%)	
Recurrence	No	5 (45.5%)	4 (14.3%)	.085
	Yes	6 (54.5%)	24 (85.7%)	
DFS (m)		54.46	17.58	.048*
OS(m)		56.37	32.26	.128

Bold*: statistically significant

### Comparison of GLUT-1 extension with prognostic variables and survival analysis

**Fig. 3:**
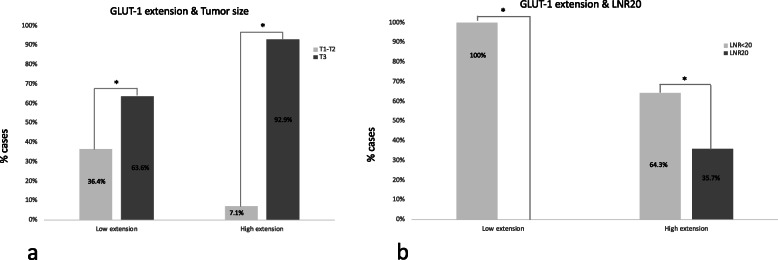
Immunohistochemistry graphs. Representing GLUT-1 extension versus **a**) tumour size and **b**) LNR20

Patients were divided into low versus high extension groups (cut off at 80% of stained cells). Regarding demographic parameters, no differences between the groups were identified. The high extension group showed a higher percentage of T3 PDAC (92.9% vs 63.6%, *p* = 0.042), and higher LNR20 (35.7% vs 0%, *p* = 0.037) (Fig. 3). Moreover, the higher extension also correlated with a higher rate of N+ (64.3% vs 27.3%, *p* = 0.072), worse tumour differentiation (42.9% vs 16.7%, *p* = 1), and higher recurrence rate (85.7% vs 54.5%, *p* = 0.085) (Table 2). Regarding survival, the high extension group showed a significant decrease in DFS (17.58 vs 54.46 months; *p* = 0.048). OS was also shorter in the high-extension group (32.26 vs 56.37 months; *p* = 0.128), without reaching significant differences (Fig. 2).

## Discussion

There has been little progress in pancreatic cancer treatment compared to cancers in other locations during recent decades [4, 21]. With such a background, it seems clinically important to find markers that can identify subgroups of patients who may benefit from aggressive therapy in an attempt to improve survival [22]. A variety of clinical and pathological factors have been reported [23]; however, there is still controversy over which ones can be used as independent predictors and the significance of their influence on patient survival [24]. Regarding pancreatic carcinoma, an immunohistochemical overexpression of GLUT-1 has been described as being associated with a worse prognosis. Our study correlates with the findings of previous studies because patients with a higher percentage of stained cells had significantly larger tumours and greater lymph node involvement. Tumour differentiation and recurrence also showed a positive trend in the high-intensity and extension groups. DFS was significantly shorter in the high-extension group and both DFS and OS seemed to be shorter in both analyses, despite not reaching significance.

The study by Lyshchik et al. [24] was one of the first to investigate the immunoreactivity of tumour cells marked with GLUT-1. The researchers divided patients into five groups, assigning points based on the percentages of stained cells; this was a negative study and the results did not show any significant correlation between GLUT-1 expression and patient survival. Another study [3], in which patients were divided using similar criteria, found significant differences regarding histological grade. Moreover, in the multivariate analysis, the stage and GLUT-1 expression were prognostic factors when stratifying the extension into < or > 50%. In this study, expression in preneoplastic lesions was also analysed; it was negative in PANIN 1 but positive in 27.8 and 43.8% of PANIN 2 and 3, respectively. Similarly, no expression was seen in low-grade IPMN while 60% of high-grade IPMN presented strong expression. These results were corroborated by Basturk et al. [2], whose study again showed a progressive increase in the expression of GLUT-1 as they analysed higher graded lesions and that not only the histological grade but also tumour size correlated to higher expression of GLUT-1. Similar results were found in two recent retrospective studies [21, 25] and one metanalysis [26] regarding bigger tumour size, a further advanced stage, lymphatic metastases, and shorter OS. It was also seen that in multivariate analysis GLUT-1 expression was an independent prognostic factor. Finally, Kurahara et al. [27] presented the first study including patients with neoadjuvant therapy whose results also support our findings, showing that those with low GLUT-1 expression displayed a better therapeutic response to neoadjuvant chemotherapy, thus, a better prognosis. However, the limitation of this study, is that chemotherapy used is only available in Japan and the population recruited in the study was Asian, which could make those results non-transposable to western countries. Due to the time the patients were initially treated, our study does not include patients with neoadjuvant treatment but a long follow-up, uncommon in this type of study, is applied in a western population and is a homogeneous sample of patients with adenocarcinoma of the head of the pancreas only, thus eliminating confounding factors.

### Limitations

The authors recognise some limitations of this study. Due to its retrospective nature and the time frame between the initial treatment and the current era, great developments and experience have been gained in the different fields, namely intensive care management, surgery, and oncology. These changes in management could have modified patient survival. Besides, a small number of patients were enrolled and for a high number of advanced cases (most of them were T3, N1, IIB, poorly differentiated, and had high extension) it could have been difficult to obtain significant differences. Moreover, subtypes other than GLUT-1 were not included. Therefore, further studies, on larger populations and with more types of GLUTs and other metabolic markers, could help to elucidate the role of these immunodiagnostic tools and the molecular mechanism underlying in the carcinogenesis of pancreatic cancer.

## Conclusion

Our study demonstrated that overexpression of GLUT-1 was related to bigger tumours and increased percentage of N+ PDAC. In terms of survival, the OS did not reach significant differences but patients with more extensive staining were found to have significantly shorter DFS. Our findings suggest that GLUT-1 could be related to higher aggressivity in PDAC, therefore, our paper supports previous studies confirming the already known idea that GLUT-1 could be a prognostic marker, identifying patients with a worse response to current therapies who could benefit from more aggressive treatments. Further prospective studies are warranted to confirm our results.

## Supplementary information

**Additional file 1.** GLUT-1 stained head of the pancreas adenocarcinoma sample. Visualized at 40 magnification, showing intraneural invasion (arrow) and perineural invasion (arrowhead).

**Additional file 2.** GLUT-1 stained head of the pancreas adenocarcinoma sample. Visualized at 40 magnification, showing germinal centres (arrow) and subcapsular metastases of peripancreatic lymph nodes (arrowhead).

## Data Availability

The data that support the findings of this study are available from Hospital Universitario de la Princesa but restrictions apply to the availability of these data, which were used under license for the current study, and so are not publicly available. Data are however available from the authors upon reasonable request and with permission of Hospital Universitario de la Princesa.

## Abbreviations

PDAC: Pancreatic ductal adenocarcinoma
GLUT-1: Glucose transporter type 1
PD: Pancreaticoduodenectomy
CT: Computed tomography
ASA: American Society of Anaesthesiologist classification
BMI: Body mass index
T: Tumour size
N: Lymph node status
LNR20: Lymph node ratio 20
R: Resection margin rate
DFS: Disease-free survival
OS: Overall survival

## References

[1] Ducreux M, Cuhna AS, Caramella C, Hollebecque A, Burtin P, Goéré D (2015). Cancer of the pancreas: ESMO clinical practice guidelines for diagnosis, treatment and follow-up. Ann Oncol.

[2] Basturk O, Singh R, Kaygusuz E, Balci S, Dursun N, Culhaci N (2011). GLUT-1 expression in pancreatic neoplasia: implications in pathogenesis, diagnosis and prognosis. Pancreas.

[3] Pizzi S, Porzionato A, Psquali C, Guidolin D, Sperti C, Fogar P (2009). Glucose transporter-1 expression and prognostic significance in pancreatic carcinogenesis. Histol Histopathol.

[4] Jemal A, Siegel R, Ward E, Hao Y, Xu J, Murray T (2008). Cancer statistics, 2008. CA Cancer J Clin.

[5] Muniraj T, Uk M, Jamidar PA, Aslanian HR (2013). Pancreatic cancer : a comprehensive review and update. Dis Mon.

[6] Lillemoe KD, Yeo CJ, Cameron JL (2000). Pancreatic Cancer: state-of-the-art Care. CA Cancer J Clin.

[7] Kondo T, Kanai M, Kou T, Sakuma T, Mochizuki H, Kamada M (2017). Impact of BRCAness on the efficacy of oxaliplatin-based chemotherapy in patients with unresectable pancreatic cancer. J Clin Oncol.

[8] Kondo T, Kanai M, Kou T, Sakuma T, Mochizuki H, Kamada M (2018). Association between homologous recombination repair gene mutations and response to oxaliplatin in pancreatic cancer. Oncotarget.

[9] Vitoratou DI, Tolia M, Liakos P, Tsoukalas N, Giaginis C, Nikolaou M (2019). Clinical value of significance of hypoxia inducible factor-1α, glucose transporter-1 and carbonic anhydrase IX in rectal cancer after preoperative chemoradiotherapy. J BUON.

[10] Osugi J, Yamaura T, Muto S, Okabe N, Matsumura Y, Hoshino M (2015). Prognostic impact of the combination of glucose transporter 1 and ATP citrate lyase in node-negative patients with non-small lung cancer. Lung Cancer.

[11] Ikeda K, Tate G, Takao S, Kitamura T, Mitsuya T (2011). Diagnostic Usefulness of EMA, IMPN3, and GLUT-1 for the Inmunocytochemical Distinction of Malignant Cells From Reactive Mesothelial Cells in Effusion Cytology Using Cytospin Preparations. Diagn Cytopathol.

[12] Yang H, Zhong JT, Zhou SH, Han HM (2019). Roles of GLUT-1 and HK-II expression in the biological behavior of head and neck cancer. Oncotarget.

[13] Rudlowski C, Moser M, Becker AJ, Rath W, Buttner R, Schroder W (2004). GLUT1 mRNA and protein expression in ovarian borderline tumors and cancer. Oncology.

[14] Boyaci C, Behzatoglu K (2018). Diagnostic value of glucose transporter 1 (glut-1) expression in nested variant of urothelial carcinoma. Turkish J Pathol.

[15] Gasinska A, Jaszczynski J, Rychlik U, Łuczynska E, Pogodzinski M, Palaczynski M. Prognostic significance of serum PSA level and telomerase, VEGF and GLUT-1 protein expression for the biochemical recurrence in prostate Cancer patients after radical prostatectomy. Pathol Oncol Res. 2020;26(2):1049–56. https://www.ncbi.nlm.nih.gov/pmc/articles/PMC7242245/. 10.1007/s12253-019-00659-4 PMC7242245 30989489

[16] Zhang TB, Zhao Y, Tong ZX, Guan YF (2015). Inhibition of glucose-transporter 1 (GLUT-1) expression reversed Warburg effect in gastric cancer cell MKN45. Int J Clin Exp Med.

[17] Kobayashi M, Kaida H, Kawahara A, Hattori S, Kurata S, Hayakawa M (2012). The relationship between GLUT-1 and vascular endothelial growth factor expression and 18F-FDG uptake in esophageal squamous cell Cancer patients. Clin Nucl Med.

[18] Krawczyk MA, Styczewska M, Sokolewicz EM, Kunc M, Gabrych A, Fatyga A (2019). Tumour expressions of hypoxic markers predict the response to neo-adjuvant chemotherapy in children with inoperable rhabdomyosarcoma. Biomarkers.

[19] Clavien PA, Sanabria JR, Strasberg SM (1992). Proposed classification of complications of surgery with examples of utility in cholecystectomy. Surgery..

[20] NCCN Hepatobiliary Cancer Panel (2019). Hepatobiliary Cancers, V.2.2019.

[21] Yang H-J, Xu W-J, Guan Y-H, Zhang H-W, Ding W-Q, Rong L (2016). Expression of Glut-1 and HK-II in pancreatic Cancer and their impact on prognosis and FDG accumulation. Transl Oncol.

[22] Ghaneh P, Kawesha A, Evans JD, John P (2002). Neoptolemos. Molecular prognostic markers in pancreatic cancer. J Hepato-Biliary-Pancreat Surg.

[23] Lim JE, Chien MW, Earle CC (2003). Prognostic factors following curative resection for pancreatic adenocarcinoma: a population-based, linked database analysis of 396 patients. Ann Surg.

[24] Lyshchik A, Higashi T, Hara T, Nakamoto Y, Fujimoto K, Doi R (2007). Expression of glucose transporter-1, hexokinase-II, proliferating cell nuclear antigen and survival of patients with pancreatic cancer. Cancer Investig.

[25] Lu K, Yang J, Li DC, He SB, Zhu DM, Zhang LF (2016). Expression and clinical significance of glucose transporter-1 in pancreatic cancer. Oncol Lett.

[26] Sharen G, Cheng H, Shi Y, Zhao J, Liu Y, Peng Y (2017). Prognostic value of GLUT-1 expression in pancreatic cancer: results from 538 patients. Oncotarget.

[27] Kurahara H, Maemura K, Mataki Y, Sakoda M, Iino S, Kawasaki Y (2018). Significance of glucose transporter type 1 (GLUT-1) expression in the therapeutic strategy for pancreatic ductal adenocarcinoma. Ann Surg Oncol.

