# Supplementary figures and images for: GLUT-1 as a predictor of worse prognosis in pancreatic adenocarcinoma: immunohistochemistry study showing the correlation between expression and survival
Source: BMC Cancer. 2020 Sep 23;20:909. doi: 10.1186/s12885-020-07409-9 (PMC7510075; doi:10.1186/s12885-020-07409-9)

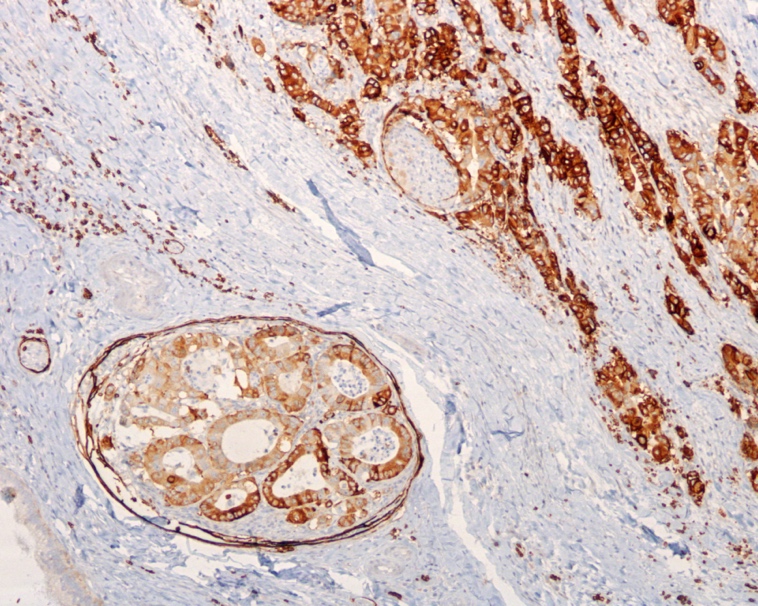

Supplement: Supplementary file 1 — Additional file 1. GLUT-1 stained head of the pancreas adenocarcinoma sample. Visualized at 40 magnification, showing intraneural invasion (arrow) and perineural invasion (arrowhead). [file 12885_2020_7409_MOESM1_ESM.docx]

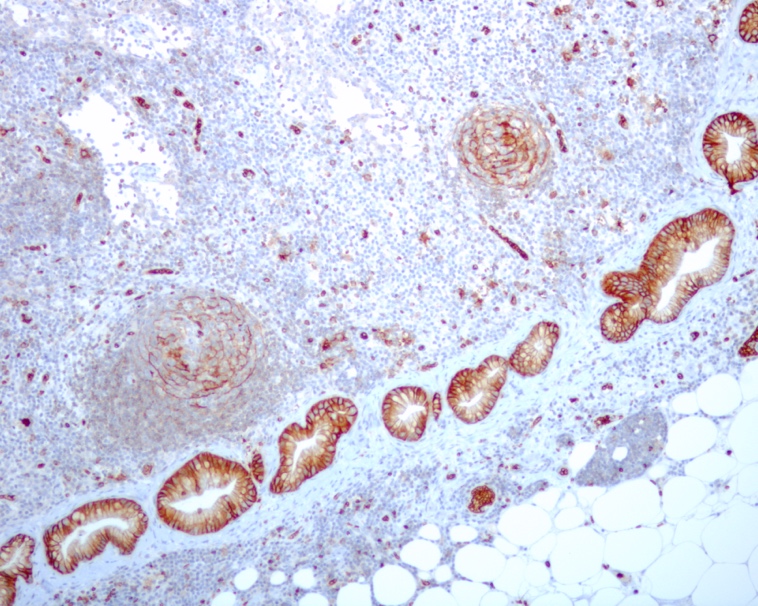

Supplement: Supplementary file 2 — Additional file 2. GLUT-1 stained head of the pancreas adenocarcinoma sample. Visualized at 40 magnification, showing germinal centres (arrow) and subcapsular metastases of peripancreatic lymph nodes (arrowhead). [file 12885_2020_7409_MOESM2_ESM.docx]
